# Electrospinning Microencapsulation of *Lactobacillus fermentum* K73 Using Gelatin as the Main Component of a Food-Grade Matrix

**DOI:** 10.3390/microorganisms11112682

**Published:** 2023-11-01

**Authors:** Arjana Serrano-Delgado, María Ximena Quintanilla-Carvajal

**Affiliations:** Universidad de La Sabana, Facultad de Ingeniería, Campus del Puente del Común, km 7 Autopista Norte de Bogotá, Chia 250001, Cundinamarca, Colombia; arajana.serrano@unisabana.edu.co

**Keywords:** probiotic, microencapsulation, electrospinning, gelatin, soy lecithin

## Abstract

This work aimed to establish the conditions that improve the viability of *Lactobacillus fermentum* K73 during and after the electrospinning process. A mixture of experimental designs were performed to select the formulation (gelatin and bacterial culture) that improves the probiotic viability after blending and under simulated gastrointestinal conditions. A Box–Behnken design was performed to improve the encapsulation yield and survival during the electrospinning process. For the Box–Behnken design, the factors were soy lecithin and bacteria culture concentration at the blend and collector distance for electrospinning. It was hypothesized that soy lecithin improved the electrospinnability, acting as a surfactant in the mixture and allowing lower voltage to be used during the process. The selected volume ratio of the gelatin (25%)/bacterial culture mixture was 0.66/0.34. The physicochemical parameters of the selected blend were in the recommended range for electrospinning. The conditions that improved the encapsulation yield and survival during electrospinning were 200 g/L of bacterial culture, 2.5% (*w*/*v*) soy lecithin, and 7 cm collector distance. The experimental encapsulation yield and survival was 80.7%, with an experimental error of 7.2%. SEM micrographs showed the formation of fibers with gelatin/bacterial culture beads. Encapsulation improved the viability of the probiotic under simulated gastrointestinal conditions compared to free cells.

## 1. Introduction

Probiotics are considered live organisms that provide health benefits for the host if they are consumed in adequate amounts while viable [1]. Currently, the development of foods enriched with probiotics represents an industrial sector with a high commercial interest and a growing market [2]. The worldwide probiotics market is predicted to reach USD 76.7 billion by 2027 [3]. The high demand for foods enriched with probiotics is related to their beneficial effects on human health and consumer awareness about nutrition and those health benefits [4]. Probiotics like *Lactobacillus fermentum*, *Lactobacillus plantarum*, *Lactobacillus acidophilus,* among others are employed to keep the organism equilibrium between beneficial and pathogenic microorganisms. Probiotics can help to restore the microbiota after antibiotic therapy through several mechanisms. One of the mechanisms is to produce antimicrobial compounds such as bacteriocins, ethanol, acetic acid, and hydrogen peroxide that cause cell death to pathogens like *Salmonella enterica*, *Clostridium difficile,* and *Escherichia coli*, among others [5,6]. Another strategy is competitive inhibition on the intestinal epithelial surface, which reduces pathogen interaction by blocking the adhering sites on the intestinal epithelial surface. Probiotics can also inhibit the growth of pathogens and microorganisms by a mechanism called competition for nutrients [7]. Probiotics can stimulate the immune system through the production of several vitamins such as B1, B2, B3, B6, B9, and B12. For example, *Lactobacillus plantarum* produces vitamin B12 extracellularly [8]. Additionally, probiotics have a positive effect on the immune system due to the stimulation of antibodies production (IgA), which plays an antimicrobial role in the presence of pathogenic microorganisms [9]. They can induce the production of antimicrobial peptides and cytokines [10]. Overall, the positive effects on human health are associated with antimicrobial, anti-inflammatory, anticancer, and anti-allergic properties [11]. Evidence of the beneficial effect of probiotics has been demonstrated with both in vivo and in vitro experiments [12]. For example, *Lactobacillus fermentum* K73 has hypocholesterolemic potential in vitro [7]. *L. fermentum* K73 produces bile salt hydrolase (BSH) (E.C 3.5.1.24). This enzyme deconjugates the bile salts, causing decreases in serum cholesterol and limiting its absorption by the intestinal lumen. Also, *L. fermentum* K73 can internalize cholesterol into the membrane [13,14].

The production of probiotics has become relevant because of the high demand worldwide. Nevertheless, maintaining probiotic viability is a challenge during the processing and consumption. The microorganism must be able to tolerate food processing, storage, and gastrointestinal conditions. The processing and storage conditions are related to a low pH, the composition of the food matrix, extreme temperatures (cold chain and thermal treatments), and osmotic pressure. Gastrointestinal conditions include low pH values, hydrolytic enzyme activity, the presence of bile salts, and osmotic stress [15,16,17,18].

One strategy to improve probiotic viability is the microencapsulation technique. Several techniques have been used for encapsulating bioactive compounds, such as spray-drying, emulsion, extrusion, spray chilling, and fluidized beds [19,20]. Those methods are performed under harsh conditions such as extreme temperature, oxidative stress, and an organic solvent environment that causes cell death [19]. Extrusion is a method that permits maintenance of cell viability. This method does not require organic solvents, severe temperatures, or pH levels. It performs well in lab settings and is less expensive. However, the product’s size, which ranges from 10 um to 5 mm, can alter the organoleptic characteristics of food [21,22]. Emulsion encapsulation (internal ionic gelation) is a different method that has also demonstrated good viable survival rates. This method is straightforward and yields beads with tiny diameters (200 nm to 1 mm). However, typical emulsions are thermodynamically unstable, unsuited for low-fat food matrices, and have a high production cost [22].

An alternative method for encapsulation is the electrospinning process [23]. This method is a fast and continuous process that operates at room temperature and allows sensitive living cells to be encapsulated [24]. Electrospinning does not involve severe conditions of temperature, pressure, or organic solvents, avoiding the negative effects on the cells compared with other techniques [25]. Maintaining viability during the encapsulation process is critical to ensuring that probiotics produce beneficial effects in consumers after they are incorporated into a food matrix. Also, this technique is an efficient way to create sub-micron or nanoscale polymer fiber, and the size is controlled by adjusting processing parameters. Size is very important as it affects the sensory properties of the food matrix. A product size greater than 30 μm will produce a gritty mouthfeel sensation, and less than 10 µm is preferred because it can reduce the detrimental sensory effects [26,27,28,29]. The application of this technique in the industrial field requires the use of food-grade wall materials. Thus, the wall materials must be degraded during human digestion, non-toxic, and authorized for use by an agency like the US Food and Drug Administration (FDA) [30,31].

Gelatin is a natural polymer approved by the FDA which has also been recognized for properties such as good biocompatibility, biodegradability, non-toxicity, easy availability, and digestibility [32,33,34,35]. Gelatin has been used in the food industry as a biodegradable packaging material and a vehicle for probiotic encapsulation [36]. Type A gelatin is produced by acid hydrolysis of collagen, which results in a structure of α-chains, β-chains, and γ-chains. The α-chains (one, two, or three) can form a double- or triple-strand structure. The β-chains and γ-chains are formed by covalent bonds between α-chains which are different from the double- or triple-strand structures that form helices stabilized by weak bonds. The triple-helix structure of gelatin confers the capacity to form fibers by electrospinning [37,38,39]. The electrospinning process must guarantee a high encapsulation yield of the probiotic bacteria. The ability of the solution to produce fibers via electrospinning is controlled by several parameters including surface tension, viscosity, and the conductivity of the solution. To improve electrospinnability, the surface tension could be reduced by the addition of surfactants to the polymeric solution [30,40]. The addition of surfactants could also increase the process stability, which decreases the dripping, facilitating the encapsulation and allowing the use of lower voltages. Also, the use of surfactants improves the dispersion of the microorganism in the mixture, allowing a continuous process and increasing the product yield of the encapsulated bacteria [40,41,42].

Some studies have aimed to obtain a probiotic encapsulated by electrospinning using different types of wall materials. For example, Diep and Schi [43] evaluated the encapsulation by electrospinning of an *E. coli* strain within alginate/poly (ethylene oxide)/polysorbate 80. Feng et al. [15] evaluated the electrospinning of *L. plantarum* within polyvinyl alcohol (PVA) and sodium alginate and obtained improved tolerance to gastrointestinal conditions. Also, Mojaveri, Hosseini, and Gharsallaoui [44] obtained electrospun fibers of *Bifidobacterium animalis* within PVA/acid acetic/chitosan and the survival in simulated gastric and intestinal fluids was improved. However, some of these materials such as PVA and PEO are expensive, and although they are food-grade approved materials, they have low absorption rates and they are recommended for use in only small concentrations [31,45].

Thus, this work aimed to establish the electrospinning conditions that maintain the viability of *L. fermentum* K73. A mixture of experimental and Box–Behnken designs were performed to improve the encapsulation yield and survival during the electrospinning process. The first design selected the optimal proportion of wall materials (bacterial culture and gelatin). The study also evaluated the effects of adding soy lecithin as a surfactant, the concentration of bacterial culture in the mixture, and the collector distance on the encapsulation yield and survival during the electrospinning process. Finally, the tolerance of the electrospun microfibers under simulated gastrointestinal conditions was evaluated.

## 2. Materials and Methods

### 2.1. Materials

Yeast extract (Oxoid Ltd., Basingstoke, UK), De Man Rogosa and Sharpe (MRS) agar and broth, peptone water (Sharlau Microbiology, Barcelona, Spain), sweet whey (11 wt% protein, 1.5 wt% fat, 69.5 wt% lactose; Marovia Lacto A.S., Czech Republic), type A gelatin (Cimpa S.A.S., Bogotá, Colombia), food-grade non-purified soy lecithin (Manuchar Colombia Cía. S.A.S., Colombia), bromelain enzyme (MP Biomedicals, Santa Ana, CA, USA), and bile salts mixture (Sigma Aldrich, Schnelldorf, Germany) were used.

### 2.2. Bacterial Strain and Culture Conditions

*Lactobacillus fermentum* K73 strain (GenBank KP784433, NCBI, Bethesda, MD, USA) was previously isolated from Suero Costeño (typical cheese of Colombia) [45]. The probiotic was conserved at −80 °C in 20% sterilized glycerol and MRS. Biomass production was performed in a 1.3 L bioreactor (Bioflo 110, New Brunswick Scientific Co., Inc., Edison, NJ, USA) with a working load of 0.8 L at 37 °C and an agitation speed of 100 rpm for 10 h. The bacteria were inoculated in the bioreactor at 10% of the volume of the working load. The culture medium was composed of 8% (*w*/*v*) milk whey and 0.22% (*w*/*v*) yeast extract; the pH was adjusted to 5.5, and the medium was sterilized at 125 °C for 15 min [45].

### 2.3. Preparation of Carrier Material

Wall material suspension was prepared by adding gelatin to distilled water at 50 °C with magnetic shaking until total dissolution and reaching a concentration of 25% *w*/*v*. The gelatin was hydrolyzed by addition of the enzyme bromelain. The gelatin hydrolysis prevents the polymeric mixture in the equipment from gelation, allowing for a continuous process. It also enables the manipulation of gelatin solutions at lower temperatures (30–35 °C) [3,46]. Enzyme solution was prepared by dissolving the bromelain at 25 °C in phosphate buffer (10 mM at pH 4.5). The gelatin solution temperature was decreased to 40 °C for adequate enzyme action. Then, 150 µL of bromelain (0.2 GDU) was added for 5 min (enzyme addition improves fiber formation). To stop the enzyme action, the solution was placed in a water bath until it reached 90 °C for 10 min. Then, the gelatin was cooled to 37 °C for mixing with the microorganism and the electrospinning process [47]. The bacterial culture was mixed with the gelatin suspension according to the mixture experimental design.

Each mixture (0.5 mL) was inoculated in 4.5 mL of MRS broth adjusted to pH 2 with 6 M HCl or supplemented with 0.3% *w*/*w* bile salts and incubated for 2 h at 37 °C. The bacterial viability (CFU/mL) was determined before and after incubation by plate counting and observing changes in the bacterial cycles (log CFU/mL) (Equation (1)).

(1) $$Bacterial\;cyles\;change=N_{final}\;\left(\mathrm{CFU}/\mathrm{mL}\right)-N_{initial}\;(\frac{\mathrm{CFU}}{\mathrm{mL}})$$

Thus, the bacterial cycle changes could show positive or negative values.

#### 2.3.1. Mixture Experimental Design to Select the Ratio of Wall Materials

Type A gelatin and the bacterial culture (whey serum, yeast extract, and the grown bacteria) were selected as the carrier materials. To select the optimal proportions of carrier materials, a mixture experimental design was made using Design-Expert V.8.1.1 software. The design evaluated seven mixtures (runs), with runs 3 and 6 using one and two repetitions (runs 7, 8, and 10, respectively), so the experimental design had 10 runs in total. The response variables were the changes in bacterial cycles (log CFU/mL). The selected blend showed the maximal tolerance to gastrointestinal conditions that was optimized using the desirability criteria.

#### 2.3.2. Mixture Characterization

Characterization of the mixtures was performed by measuring viscosity, surface tension, conductivity, and pH. The flow behavior of the mixture and viscosity were measured by a Modular compact rheometer with parallel-plate geometry (PP50) (Anton Paar MCR-502, Hertford, UK) [48]. The flow curve was obtained with a shear rate between 100 and 1000 s^−1^ at 37 °C. All the mixtures exhibited Newtonian behavior. Average viscosity was determined over the shear rate range of 100 to 1000 s^−1^. The surface tension of the mixtures was measured by a Sigma 700 tensiometer (Attension, Espoo, Finland) equipped with a Wilhelmy plate and the sample was heated at 37 °C before the measurement. Around 5 mL of the sample was placed in a small glass vessel for measuring the surface tension [6]. pH was measured using 850 SI-Analytics (Thermo Fisher Scientific, Houston, TX, USA) equipment and conductivity was determined using an HD 2306.0 conductivity meter (Delta OHM). All measurements were performed in triplicate and reported as the mean ± standard deviation (SD).

### 2.4. Improvement in Probiotic Viability during the Electrospinning Process through Box–Behnken Design

Three factors were selected to improve the viability of the probiotic during the electrospinning process. The factors were the bacterial culture (g/L), the collector distance (cm), and the percentage of soy lecithin (*w*/*v*) added as a surfactant. The culture was centrifuged for 30 min at 6000 rpm and 4 °C, and then the precipitate was collected and diluted in 0.1% (*w*/*v*) peptone water. A Box–Behnken experimental design was performed with three factors and three levels using Design-Expert V.8.1.1 software. The design evaluated 11 treatments (runs), with the third run using four repetitions (runs 6, 8, 11, and 15, respectively), so the experimental design had 15 runs in total. The response variable was the encapsulation yield and survival (log CFU/mL) after the encapsulation process (Equation (2)).

(2) $$Encapsulation\;yield\;and\;survival\;\%=\frac{{\left(logCFU/mL\right)}_{N1}}{{\left(logCFU/mL\right)}_{N2}}\times 100$$

where *N*1 indicates the number of viable bacteria released from the fibers and *N*2 is the number of viable bacteria in the mixture [49]. The encapsulation yield measures the efficacy of entrapment and the survival of viable cells during electrospinning [9].

#### 2.4.1. Electrospinning Process

The microencapsulation procedure was performed with the optimal mixture using Fluidnatek^®^ LE-100 equipment (BioInicia, Valencia, Spain) fitted with a capillary tube (∅ = 1/16″ outer diameter, OD), plastic syringe (20 mL capacity), syringe pump, high-voltage source, and a flat collector. The optimal mixture was maintained at 42 °C, introduced via sterile syringe, and pumped at a constant flow rate of 5 mL/h through a stainless steel needle. Three collector distances were used (7, 8, and 9 cm, following the design) and a voltage of 13.6–17.6 kV was applied until a Taylor cone was observed.

#### 2.4.2. Cell Count

The cell count was performed by plate counting in MRS agar before and after the microencapsulation process following the method of López-Rubio et al. [50] with modifications. The cell count of the mixture was performed by serial dilutions in 0.1% (*w*/*v*) peptone water. For the fiber cell count, the sample was diluted in phosphate buffer (pH 6.6, 0.1 M) and the solution was placed in a water bath at 42 °C for 1 h. The temperature of 42°C was tested as part of this study and shown to have no effect on probiotic viability. Then, serial dilutions in 0.1% (*w*/*v*) peptone water were prepared. The final number of colony-forming units per milliliter (CFU/mL) was determined based on the number of colonies multiplied by the inverse dilution factor.

#### 2.4.3. Scanning Electron Microscopy (SEM)

The morphology of the microfibers was observed through SEM (LYRA3, TESCAN, Kohoutovice, Czech Republic). The equipment was operated at an acceleration voltage of 10 kV. The samples were covered with gold. Then, the fibers were attached to carbon tape fixed to a metallic pin and observed at 10,000× and 45,000×. The diameter and length of the beads/fibers were calculated by measuring 50 to 80 fibers from two SEM micrographs using ImageJ 1.54 D software. Diameter and length distribution histograms were plotted with the data obtained.

### 2.5. Survival Percentage of Encapsulated L. fermentum K73 during Simulated Gastrointestinal Conditions

Survival of the probiotic encapsulated under simulated gastrointestinal conditions (pH 2.0 and 0.3% (*w*/*w*) bile salts) was determined compared with that of free cells. The bacterial survival percentage was determined using Equation (3) [51]

(3) $$Survival\%=\frac{{\left(logCFU/mL\right)}_{final}}{{\left(logCFU/mL\right)}_{initial}}\times 100$$

### 2.6. Statistical Analysis

The mixture and Box–Behnken designs were performed using Design-Expert software (version 8.1.0, Stat-Ease Inc., Minneapolis, MN, USA). The significance test of the designs was performed by analysis of variance (ANOVA) with a confidence level of 95%. The coefficient of determination (R^2^) was used to evaluate the fit of the measurements to the regression models. Optimization of the response used the numerical optimization technique of Design-Expert software (version 8.1.0, Stat-Ease Inc., Minneapolis, MN, USA) and desirability criteria. Tukey’s test was performed to assess multiple comparisons between the means of the blends’ physical parameters. The correlation between physical parameters (viscosity, surface tension, pH, and conductivity) and the mass fraction of components of the blend was evaluated by a Pearson correlation test with Matlab R2023a software.

## 3. Results

### 3.1. Characterization of Polymeric Solutions of the Mixture Experimental Design

The physical features of polymeric solutions influence their electrospinning ability. The surface tension, conductivity, and viscosity observed in this study are in the ranges reported by Ricaurte and collaborators [47] for performing the electrospinning process (Table 1). The surface tension ranged from 19 to 70 mN/m, electrical conductivity from 0.35 to 7.2 mS/cm, and the viscosity was between 8.5 and 56 mPa·s [47]. The exception was mixture 1, which presented low viscosity that was not appropriate for the electrospinning process despite its high conductivity. The mixture (ratio of gelatin to culture) had a significant effect (*p* < 0.05) on the three parameters mentioned.

The surface tension of the blend is a crucial factor for electrospinning. During the electrospinning process, the electric field applied must overcome the surface tension of the polymeric solution droplet for a charged jet to be ejected and allow the production of fibers [52]. Also, the electrical conductivity influences elongation of the jet during the process and the fiber morphology. A polymeric solution with low viscosity cannot form a jet in the electrical field, favoring the formation of beads instead of fibers [53]. The physical parameters of the selected mixture were: conductivity of 1.93 ± 0.03 mS/cm, pH of 4.77 ± 0.08, surface tension of 44.3 ± 1.9 mN/m, and viscosity of 35.3 ± 4.87 mPa·s.

As seen in Table 2, the mass fractions of bacterial culture and gelatin in the blend are correlated with pH, viscosity, and conductivity according to the Pearson correlation test (*p* < 0.05). When the mass fraction of gelatin in the blend was increased, the pH and viscosity increased. Also, when the mass fraction of culture containing grown bacteria in the blend was increased, the pH and viscosity decreased, and conductivity increased. The reduction in pH values could be indicative of the bacterial concentration. Lactic acid bacteria produce organic acids that are products or intermediates of metabolic pathways [15,54]. That would explain the increase in pH when the mass fraction of gelatin was greater than that of the bacterial culture. Conductivity can be indicative of the bacterial concentration. Škrlec et al. [55] reported that the addition of *L. plantarum* to the polymeric solution for the electrospinning process increased the electrical conductivity from 2.0 to 4.8 mS/cm due to extracellular proteins and ions from the probiotic and the culture medium. The rheological properties such as viscosity are influenced by the mass fraction of gelatin. The increment of viscosity in the blend is due to an increase in protein–protein interactions caused by more gelatin molecules per volume and to the interaction of gelatin and milk proteins [56,57].

The changes of the bacterial cycles under bile salt conditions were correlated with the ratio of gelatin to bacterial culture. Thus, the value of the bacterial cycles was negative when the mass fraction of bacterial culture in the blend was increased (Table 2). Gelatin can provide a physical barrier to protect the cells under simulated intestinal conditions. Also, the intermolecular interaction between the wall materials protects the microorganism from simulated gastrointestinal conditions [58]. Hence, at a lower mass fraction of gelatin, there were fewer protein–protein interactions too. As mentioned earlier, there is a relation between conductivity and bacterial concentration. Changes in the bacterial cycles under bile salt conditions decreased when the mass fraction of the culture was increased. As conductivity increased, the bacterial cycles change under bile salt conditions decreased. This could happen because high conductivity is related to higher bacteria mass fraction compared with gelatin mass fraction; thus, there was less gelatin available to protect the probiotic under bile salt conditions.

### 3.2. Formulation of Carrier Material

Encapsulation provides a micro-environment in which the probiotic is protected. The wall materials selected must efficiently protect the probiotic to maintain a high degree of viability. Thus, the wall materials must protect the microorganism from processing, storage, and gastrointestinal conditions [21,22]. In this study, the wall materials selected were gelatin and bacterial culture (Table 3).

The microencapsulation process starts with mixture of *L. fermentum* K73 with the wall material. In this study, the mass fractions of gelatin and bacterial culture influenced bacterial growth after mixing. After blending, the response of changes in bacterial cycles was fitted to a quadratic model. The model was significant (*p* < 0.05 and R^2^ = 0.94, Table 4) with a non-significant lack of fit (*p* > 0.05) for the response variable. The interaction of bacterial culture and gelatin was significant (*p* < 0.05). The changes in bacterial cycles after mixing ranged from −0.08 to 0.19. An increase in bacterial counts indicates that the microorganism is metabolically active. The gelatin and the culture medium (milk whey) can be used as the nitrogen and carbon source for microorganism growth. Type A gelatin is produced by acid hydrolysis of collagen and the result is a mixture of polypeptides [32]. Solikhin, Mustopa, and Putranto [59] reported that *L. casei* hydrolyzes gelatin. Also, free amino acids can be generated by prolonged hydrolysis of collagen and hydrolysis of gelatin by the enzyme bromelain [26,27]. Amino acids such as leucine and serine stimulate the growth of *L. plantarum* [60]. Shu et al. [61] reported that leucine and arginine promote the growth of *L. bulgaricus*. Moreover, milk whey is rich in proteins, lactose, minerals, and oligosaccharides, which are useful for bacterial growth. *L. fermentum* uses lactose as a carbon source [46].

The wall material mass fraction influenced the loss of bacterial viability under simulated gastrointestinal conditions. The response variable of bacterial cycle changes under gastric pH and bile salt conditions fitted to a quadratic model. The results in Table 4 show that the models were significant (*p* < 0.05, R^2^: 0.88 for gastric pH and R^2^: 0.74 for bile salts) with a non-significant lack of fit (*p* > 0.05). The interaction of the culture medium with the grown bacteria and gelatin was significant (*p* < 0.05, Table 4) for survival under simulated gastrointestinal conditions.

It was hypothesized that the protective effect of wall material on *L. fermentum* K73 could be related to the interactions between gelatin, milk whey, and cells. Those interactions could form a physical barrier that protects the microorganism from simulated gastrointestinal conditions. Gelatin can interact with milk whey by hydrogen bonds which are formed between amino groups of the gelatin chain and the hydroxyl group of lactose from milk whey [62]. Also, the composition of the cell surface (proteins such as pili and polysaccharides) confers adhesive properties to molecules through intermolecular interactions such as steric hindrance, electrostatic interactions, Van der Waals forces, and hydrogen bonds [62]. Electrostatic interactions occur between bacterial cells that have a negative surface charge and type A gelatin (cationic polymer) [63,64]. Additionally, Burgain et al. [62] reported that denatured whey proteins interact specifically with the surface of *L. rhamnosus* GG cells through the pili.

The optimal volume ratio of the gelatin/bacterial culture mixture was 0.66/0.34, with a desirability value of 0.861. That mixture maximizes the bacterial counts after mixing under gastric (pH 2) and intestinal conditions (bile salts). The bacterial cycle changes predicted by the model were 0.189, −0.551, and −1.228 log CFU/mL, respectively. Experimentally, the changes in bacterial cycles after mixing, under gastric and intestinal conditions, were 0.199 ± 0.039, −0.495 ± 0.069, and −1.326 ± 0.046, with an experimental error of 5.30%, 10.1%, and 8.06%, respectively.

### 3.3. Cell Counting Process: Release of Probiotics from Electrospun Fibers

In a cell counting process, complete probiotic release is a crucial step [65]. Thus, complete release of probiotics from fibers must be guaranteed before evaluation of the electrospinning process. Viable cells were released from the fibers at 42 °C with phosphate-buffered solution (pH 6.6). In the probiotic release process, it was necessary to melt the fibers. The pH, gelatin concentration, and addition of milk whey proteins influenced the melting temperature. For example, the melting temperature is 27.5 °C for a gel of gelatin (1%) and whey protein (pH 6.6) [66]. In this study, the temperature was higher because the blends contained a higher concentration of gelatin. A high gelatin concentration leads to a shorter distance between gelatin coils and the presence of strong and more abundant junction zones [38].

**Figure 1:**
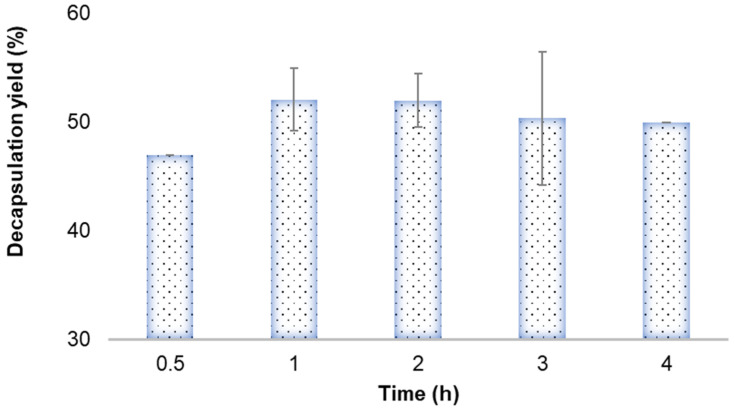
Probiotic release yield after the electrospinning process in the time.

Thirty minutes is not enough time for complete release of the probiotic from the fibers (Figure 1). The release yield was higher after 1 h in the water bath at 42 °C, and it was maintained for 2 h. There was a slight reduction in the probiotic release yield in the third and fourth hours (1.64% and 2.0%, respectively). The time of 1 h was selected for the probiotic release process. This result shows that the viability of the probiotic could be kept until the fourth hour at 42 °C. According to the INFOGEST 2.0 digestion model, the digestion process time is approximately 4 h. The time in this model is based on available physiological data [67]. Hence, probiotic viability could be maintained until the intestinal phase, but tolerance of the probiotic to all gastrointestinal conditions must be evaluated.

### 3.4. Improvement of Encapsulation Yield in the Electrospinning Process

A Box–Behnken design was performed to improve the encapsulation yield and survival during the electrospinning process (Table 5).

The response of encapsulation yield and survival was fitted to a quadratic model. The model was significant (*p* < 0.05 and R^2^ = 0.899; Table 6) with a non-significant lack of fit (*p* > 0.05) for the response variable. The addition of soy lecithin (SL) to the blend had a significant effect on encapsulation yield and survival after the electrospinning process (*p* < 0.05).

Considering that SL is an amphoteric surfactant, the hypothesis is that it reduces surface tension in the blend. Reducing the surface tension allows the application of a lower voltage in the electrospinning process and improves the bacterial survival [25]. In this study, the SL in the blend acted as a surfactant because the surface tension was reduced from 40.1 to 28.1 ± 1.01 mN/m. Also, in the blend without surfactants, the voltage was under 18 kV, and in the mixture with surfactants it was under 17 kV. Run 9 (18 kV, without surfactants) used a higher voltage than run 7 (16.4 kV, 5.0% (*w*/*v*) SL) (Table 5). However, the reduction in the voltage was not significant enough to improve the bacterial survival during the process.

**Figure 2:**
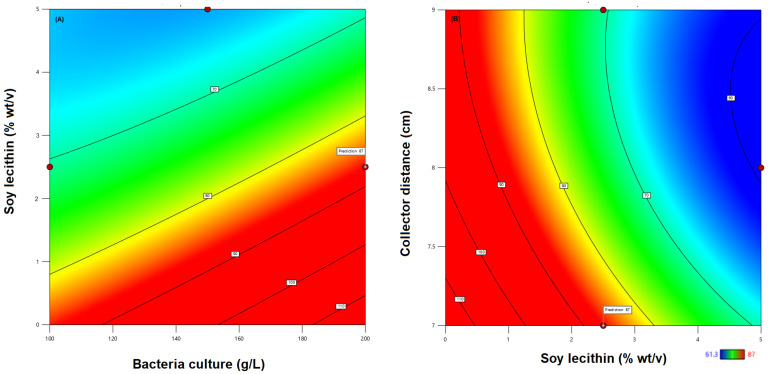
Contour graphs for the encapsulation yield and survival showing the interaction of surfactant and bacteria culture (**A**), and collector distance and surfactant concentration (**B**).

According to the results shown in Figure 2, a high encapsulation yield and survival was obtained below 2.5% (*w*/*v*) SL; a higher SL concentration reduced the encapsulation yield and survival. When the concentration of surfactants was high, the solution surface began to be saturated with free surfactant molecules, and the formation of free micelles began. Thus, the interaction between the surfactants and polymer was saturated [43,68]. This condition could avoid proper homogenization of the blend and influence the quality of the electrospinning process.

It has been shown that phospholipids can play a role in improving the viability of probiotics. The total content of phospholipids in SL is about 48% [69]. Aro et al. [70] showed that oat polar lipids (composed of phospholipids and glycolipids) protect *B. breve* in a phosphate buffer. Zhuang et al. [71] showed that SL increases the viability of *L. acidophilus* and *B. lactis* grown in MRS broth. Donthidi et al. [72] showed greater viability of *L. casei* after freeze-drying on increasing the lecithin concentration (0.25% to 4% *w*/*v*) compared with the treatment without the surfactant.

The concentration of bacterial culture and the collector distance had no significant effect on encapsulation yield and survival after the electrospinning process. The interaction of the factors had no significant effect. The expected pattern was a higher probiotic encapsulation yield and survival in blends with a high bacterial culture concentration and a larger collector distance in the electrospinning process. The short collector distance reduces solvent evaporation and generates beaded fibers, which are usually considered poor quality [73,74]. However, we expected to produce fibers with beads because of the probiotic encapsulation. Also, more protection of the cells was expected in this study due to interactions between milk whey and the cells. The hypothetical interactions were between sugar (from milk whey) and phospholipids (from bilayer) through hydrogen bonds. The macromolecules on the cell surface can interact with proteins through electrostatic and hydrophobic interactions. It has been reported that whey proteins can form a coating on the cells due to their film-forming properties [58,75].

The viability of the probiotic improved with 200 g/L of bacterial culture, 7 cm collector distance, and 2.5% (*w*/*v*) SL in the blend, achieving a desirability value of 1.00. Those conditions maximized the encapsulation yield and survival after the electrospinning process. The model predicted an encapsulation yield and survival of 87% under those conditions. The experimental encapsulation yield and survival was 81%, with an experimental error of 7.3%.

### 3.5. Morphological Characteristics

**Figure 3:**
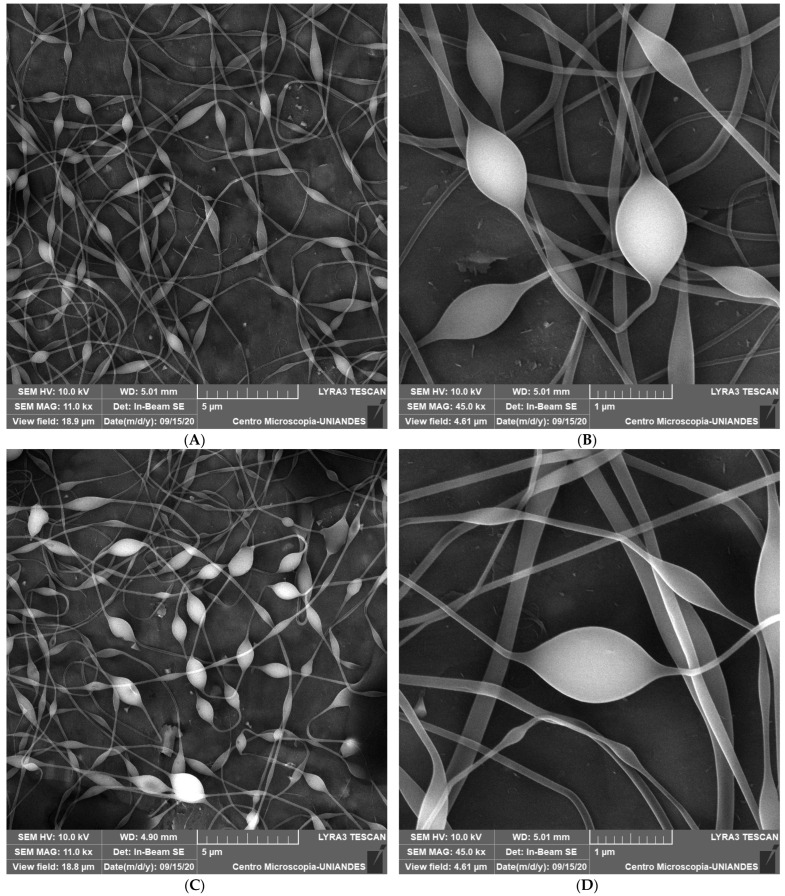

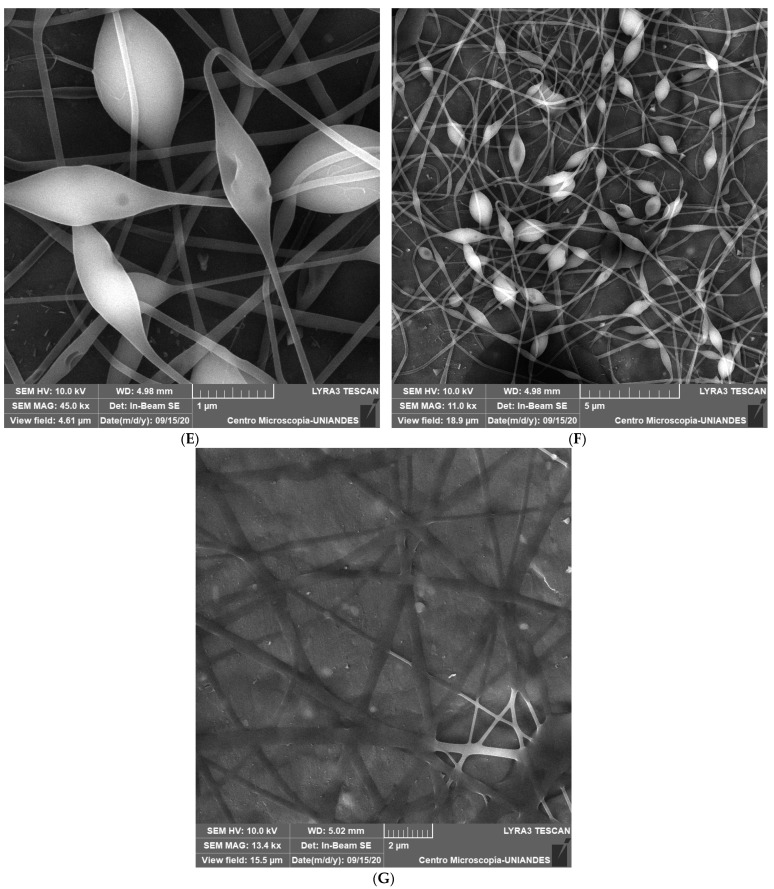
SEM micrographs of encapsulated *Lactobacillus fermentum* K73 without SL (**A**,**B**), 2.5% wt/v of SL (**C**,**D**), 5.0 % wt/v of SL (**E**,**F**), and gelatin with 2.5 % wt/v of SL (**G**).

The morphology of the encapsulated microorganism is shown in Figure 3. The fibers presented a beaded morphology with several beads in the same fiber. Similar results were reported by Feng et al. [15], who found that the addition of probiotic cells into the spinning solution resulted in beaded fiber morphology. In food products, the generation of micro-beads in fibers can break down the product and release the probiotic on mastication [43]. For that reason, in future works, the formation of beads needs to be addressed. In addition to a short collector distance, factors such as a low polymer concentration, slow solution flow rate, low applied voltage, and low surface tension can cause beading [43]. In this study, the electrospinnability of the polymeric solution (bacterial culture, gelatin, and SL) is given by gelatin due to its structure [45]. The concentration of the polymeric solution influences the morphology of the fibers; a fiber morphology is produced at the concentration for which molecular chain entanglement is sufficiently high to prevent jet break-up [76]. Thus, an increment of gelatin ratio in the blend can generate bead-free fibers. Ricaurte et al. [47] reported that the ionization of gelatin amino acids with acetic acid improves the electrospinning process to obtain defect-free fibers.

**Figure 4:**
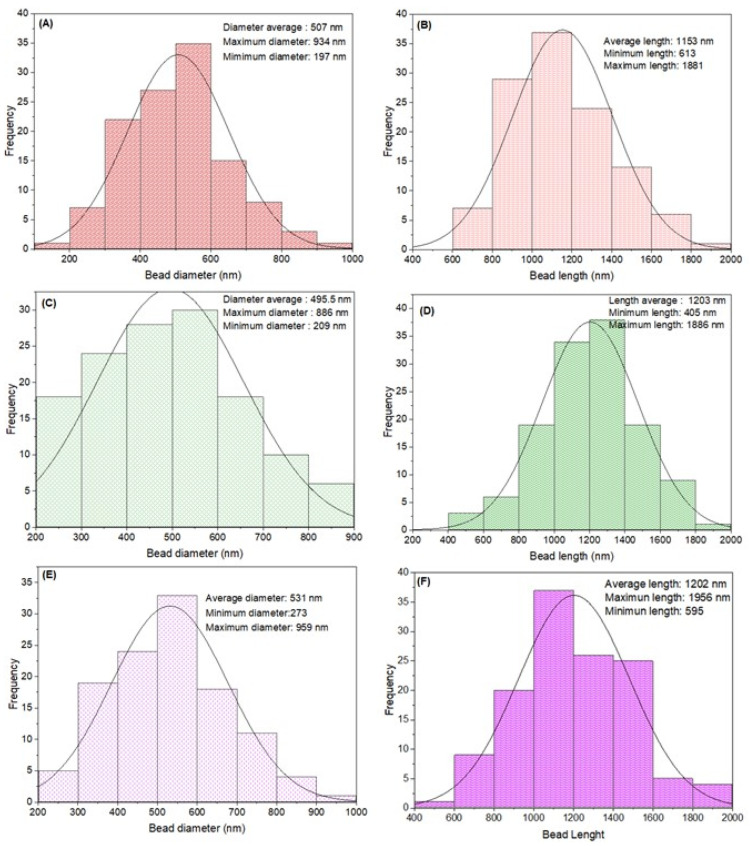
Diameter and length distribution histogram of *Lactobacillus fermentum* K73 fibers without SL (**A**,**B**), 2.5 % wt/v of SL (**C**,**D**), 5.0 % wt/v of SL (**E**,**F**).

We hypothesized that the probiotic cells were localized in the beads of the fibers due to their size. The average diameter of the beads for the blends without SL, with 2.5% (*w*/*v*) SL, and with 5.0% (*w*/*v*) SL was 507 ± 143, 496 ± 146, and 531 ± 137 nm, respectively. The average length of the beads was 1153 ± 252, 1203 ± 274, and 1202 ± 280 nm, respectively (Figure 4). Species of the genus Lactobacillus have a length of between 1000 and 1500 nm and a diameter from 700 to 1000 nm [77]. In this study, the bead length was in that range, but the diameter was slightly lower. The slight reduction in bacterial diameter could be related to the diameter of the fibers obtained, which may reduce the bacterial size. In this study, the fiber diameter was between 45 and 185 nm, and the average diameter was 110 ± 25 nm. The diameter of the fiber obtained was smaller than the bead size within the fibers. Mojaveri et al. [44] obtained *B. animalis*/chitosan/PVA fibers with a diameter of 117 nm. Feng et al. [15] produced fibers for encapsulation of *L. plantarum* with a greater diameter (270 nm) compared with that in our study.

Fiber quality was influenced by surfactant concentration. As shown in Figure 3E,F, when the surfactant concentration in the blend was high, the fibers presented pores. Porosity is an explanation for why the efficiency yield and survival at 5% (*w*/*v*) SL was lower (Figure 2). Porosity can cause fast diffusion of moisture and other fluids through the fibers. This condition reduces physical protection against unfavorable environmental factors such as extreme pH values, hence affecting the release and protection of probiotics inside [20,78]. However, some fibers obtained were porous when the blend contained 2.5% (*w*/*v*) SL, but to a lesser degree compared with 5% (*w*/*v*) SL.

### 3.6. Tolerance of Encapsulated and Non-Encapsulated Probiotic to Simulated Gastrointestinal Conditions

**Figure 5:**
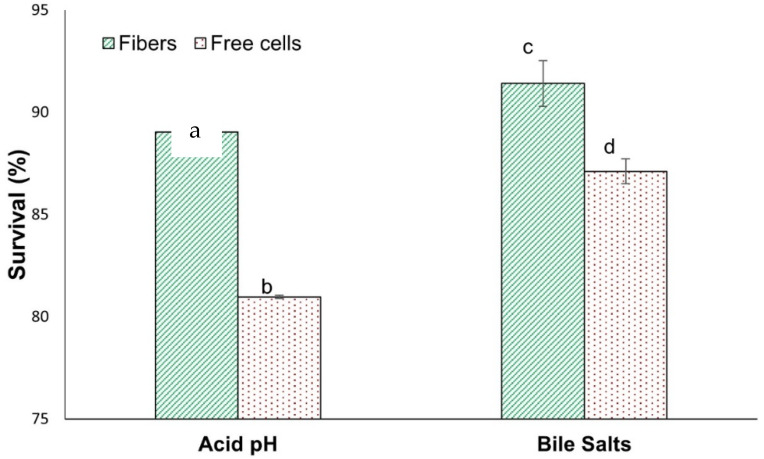
*L. fermentum* K73 encapsulated and unencapsulated survival under exposure to acid pH and bile salts. Mean value ± standard deviation of at least three independent measurements. Means that do not share a letter at the bars of acid pH and bile salts separately are significantly different (*p* < 0.05).

The viability of encapsulated and non-encapsulated *L. fermentum* K73 was evaluated after exposure to simulated gastrointestinal conditions (acid pH and bile salts). Cells incorporated into fibers showed a higher degree of survival in simulated gastric conditions than free cells (Figure 5). There were statistically significant differences between the survival of encapsulated and non-encapsulated cells under acid pH and bile salt conditions (*p* < 0.05). Therefore, the encapsulation by electrospinning satisfactorily protected *L. fermentum* K73 from simulated gastrointestinal conditions. Also, under storage conditions, the free cell viability was reduced by 27% and the encapsulated cells did not show a reduction in viability in 10 days. Free cells were susceptible to relative humidity, acid pH, and the concentration of bile salts. It appears that encapsulation with wall materials acted as a physical barrier to protect the cells from simulated gastrointestinal conditions.

Similar results have been reported by Mojaveri et al. [44] who evaluated the encapsulation of *B. animalis* by electrospinning and obtained higher survival rates under simulated gastric and intestinal conditions compared to free cells. Feng et al. [15] found that the viability of *L. plantarum* fibers was reduced by 1.06 CFU/mL under simulated gastrointestinal conditions compared with free cells, for which the reduction was 2.6 CFU/mL.

As mentioned earlier, the addition of SL had a positive influence on the encapsulation yield and survival. The SL had a positive influence, protecting the cells from simulated gastrointestinal conditions at the selected concentration (2.5%). SL can increase cell hydrophobicity, retain cell integrity, and improve the stability of the cell surface structure; thus, the addition of this surfactant avoids the damage to the cell structure caused by the presence of bile salts [79]. Bollom et al. [80] reported that probiotics (*L. acidophilus* and *B. lactis*) in bigels with phospholipids survive better during digestion. Also, a bigel of *L. acidophilus* with SL had greater viability (7–20%) than a bigel without SL under gastric and intestinal conditions. Hu et al. [79] reported that the addition of SL to a culture of probiotic strains (*L. plantarum*) improved their ability to survive in a bile salt environment. Additionally, SL can have a protective effect during storage. Zhuang et al. [71] found that an SL-based oleogel emulsion incorporating *L. acidophilus* and *B. lactis* enhanced probiotic survival after 42 days of storage.

## 4. Conclusions

Encapsulation of the probiotic by electrospinning was successful and enhanced the tolerance of the probiotic to simulated gastrointestinal conditions. The use of food-grade materials in the encapsulation process showed an attractive outcome for the functional food sector. The wall material ratio influences the viability of the probiotic under acidic pH and bile salt conditions. The protective effect on the probiotic could be due to the interaction between gelatin, milk whey, and cells. The addition of SL to the blend reduced the surface tension, allowing use of a lower voltage during electrospinning. This improved both the encapsulation yield by electrospinning and the cell tolerance to simulated gastrointestinal conditions. However, fiber quality was affected at higher SL incorporation rates due to the saturation of the surfactant on the blend. We have demonstrated that the use of electrospinning with food-grade materials as an encapsulation process is a viable approach because it was successful in improving the tolerance to simulated gastrointestinal conditions compared with free cells. The fiber wall materials acted as a physical barrier, protecting the cells from the stressful conditions while still being food-grade and digestible.

## Figures and Tables

**Table 1:** Characterization of polymeric solutions: surface tension, viscosity, conductivity, and pH *.

Mixture	Ratio		Surface Tension (mN m^−1^)	Viscosity (mPa s^−1^)	Conductivity (mS cm^−1^)	Ph
	Gelatin	Culture				
1	0.20	0.80	42.9 ± 0.03 ^a^	4.80 ± 0.28 ^d^	4.88 ± 0.25 ^a^	4.10 ± 0.06 ^a^
2	0.35	0.65	39.3 ± 0.025 ^b^	7.90 ± 0.70 ^d^	4.10 ± 0.17 ^b^	4.20 ± 0.00 ^a^
3	0.40	0.60	40.8 ± 1.76 ^ab^	19.55 ± 0.49 ^c^	3.76 ± 0.08 ^b^	4.40 ± 0.11 ^a^
4	0.50	0.50	42.0 ± 0.62 ^ab^	23.90 ± 0.56 ^c^	1.95 ± 0.03 ^c^	4.40 ± 0.05 ^a^
5	0.60	0.40	42.6 ± 0.98 ^a^	33.95 ± 2.19 ^b^	1.73 ± 0.04 ^c^	4.40 ± 0.04 ^a^
6	0.65	0.35	42.7 ± 1.08 ^a^	34.10 ± 1.6 ^b^	1.58 ± 0.00 ^c^	4.50 ± 0.19 ^a^
7	0.80	0.20	42.5 ± 0.82 ^a^	48.58 ± 1.38 ^a^	1.92 ± 0.07 ^c^	4.90 ± 0.02 ^a^

* Mean value ± standard deviation of at least three independent measurements. Means that do not share a letter in the same column are significantly different (*p* < 0.05).

**Table 2:** Pearson correlation tests of physical properties, proportion of wall material, and bacterial count changes under bile salts.

Correlation	Pearson Correlation Coefficient	*p*-Value
A/B and pH	0.937	0.002
A/B and viscosity	0.915	0.004
B/A and conductivity	0.916	0.004
B/A and pH	−0.764	0.045
B/A and viscosity	−0.858	0.013
B/A and bacterial cycles change under bile salts	−0.830	0.021
Conductivity and bacterial cycles change under bile salts	−0.953	0.001

Correlation is significant at the 0.05 level. Viscosity (mPa s^−1^), conductivity (mS cm^−1^), bacterial cycle change (log CFU/mL). A: Mass fraction of gelatin, B: Mass fraction of culture with grown bacteria.

**Table 3:** Mixture experimental design to select the optimal wall materials.

Run	Factor wt% (Wall Material)		Response Variable: Bacterial Cycles Change log (CFU/mL)		
	Gelatin [A]	Culture [B]	After Mixture	Gastric pH	Bile Salt
1	0.40	0.60	0.04	0.09	−1.87
2	0.65	0.35	0.19	−0.16	−1.25
3	0.20	0.80	−0.07	−3.18	−2.48
4	0.60	0.40	0.10	−0.56	−1.11
5	0.50	0.50	0.05	0.01	−1.22
6	0.80	0.20	−0.05	−2.05	−1.39
7	0.20	0.80	−0.06	−3.20	−1.98
8	0.80	0.20	−0.08	−3.15	−1.70
9	0.35	0.65	0.15	−1.59	−1.59
10	0.80	0.20	−0.08	−2.01	−1.55
*p*-value			0.0008	0.0005	0.0080

**Table 4:** Analysis of variance (ANOVA) for the mixture design and regression equation for the response variables (Bacterial cycle changes after mixture and under gastric pH and salt bile conditions).

Source	Change in Bacterial Cycles														
	After Mixture					Gastric pH					Bile Salts				
	SS *	Df **	Mean Square	F-Value	*p*-Value	SS *	Df **	Mean Square	F-Value	*p*-Value	SS *	Df **	Mean Square	F-Value	*p*-Value
Model	0.09	4	0.02	34.5	0.0008	14.6	2	7.29	27.6	0.0005	1.39	2	0.69	10.4	0.0080
Linear mixture	6.19 × 10^−4^	1	6.19 × 10^−4^	0.95	0.3740	0.280	1	0.280	1.05	0.3399	0.59	1	0.59	8.89	0.0204
AB	9.07 × 10^−3^	1	9.07 × 10^−3^	13.9	0.0135	14.3	1	14.3	54.1	0.0002	0.80	1	0.80	11.90	0.0107
AB(A-B)	2.25 × 10^−3^	1	2.25 × 10^−3^	3.46	0.1220										
AB(A-B)^2^	0.02	1	0.023	34.7	0.0020										
Residual	3.25 × 10^−3^	5	6.50 × 10^−4^			1.85	7	0.260			0.47	7	0.07		
Lack of fit	2.60 × 10^−3^	2	1.30 × 10^−3^	6.00	0.0894	1.01	4	0.250	0.91	0.5543	0.29	4	0.07	1.28	0.4376
Pure error	6.50 × 10^−4^	3	2.17 × 10^−4^			0.84	3	0.280			0.17	3	0.06		
Corr. Total	0.09	9				16.43	9				1.86	9			
R^2^	0.96					0.88					0.74				
Equation	N (log CFU/mL) = −0.045358 ∗ A − 0.040809 ∗ B + 1.73313 × 10^−3^ ∗ A ∗ B + 1.39291 × 10^−6^ ∗ B ∗ A ∗ (A − B) + 2.54879 × 10^−7^ ∗ A∗ B ∗ (A − B)^2^					N (log CFU/mL Log) = −0.070443 ∗ A − 0.084910 ∗ B + 3.09535 × 10^−3^ ∗ A ∗ B					N (log CFU/mL) = 0.024460 ∗ A − 0.036966 ∗ B + 7.29884 × 10^−4^ ∗ A ∗ B				

A: Gelatin, B: Culture, * Sum of squares, ** Degrees of freedom.

**Table 5:** Box–Behnken experimental design to select conditions that improve encapsulation yield and survival during the electrospinning process.

	Factors			Response Variable Encapsulation Yield and Survival (%)
Run	Bacteria Culture (g/L)	Soy Lecithin (%wt/v)	Collector Distance (cm)	
1	150	5.0	9.0	65
2	200	2.5	9.0	67
3	150	2.5	8.0	66
4	100	2.5	7.0	73
5	200	2.5	7.0	87
6	150	2.5	8.0	69
7	100	5.0	8.0	65
8	150	2.5	8.0	70
9	100	0.0	8.0	75
10	100	2.5	9.0	71
11	150	2.5	8.0	68
12	150	5.0	7.0	63
13	200	5.0	8.0	63
14	150	0.0	9.0	86
15	150	2.5	8.0	61

**Table 6:** Analysis of variance (ANOVA) for the Box–Behnken design and regression equation for the response variable (encapsulation yield and survival).

	Sum of Squares	DF *	Mean Square	F-Value	*p*-Value
Model	752.71	9	83.63	4.98	0.0459
[A]-Bacteria culture	76.80	1	76.80	4.57	0.0855
[B]-Soy lecithin	450.67	1	450.67	26.83	0.0035
[C]-Collector distance	76.80	1	76.80	4.57	0.0855
AB	78.11	1	78.11	4.65	0.0835
AC	72.25	1	72.25	4.30	0.0928
BC	24.11	1	24.11	1.44	0.2846
A^2^	17.14	1	17.14	1.02	0.3587
B^2^	70.08	1	70.08	4.17	0.0965
C^2^	89.05	1	89.05	5.30	0.0695
Residual	83.97	5	16.79		
Lack of Fit	37.50	1	37.50	3.23	0.1468
Pure Error	46.47	4	11.62		
R^2^	0.899				

* DF: Degree of freedom.

## Data Availability

The datasets used and/or analyzed during the current study are available from the corresponding author on request.
